# Association of Dietary Inflammatory Index With Depression and Suicidal Ideation in Older Adult: Results From the National Health and Nutrition Examination Surveys 2005–2018

**DOI:** 10.3389/fpsyt.2022.944154

**Published:** 2022-07-05

**Authors:** Yingqi Xiao, Wei Huang

**Affiliations:** ^1^Department of Pulmonary and Critical Care Medicine, Dongguan Tungwah Hospital, Dongguan, China; ^2^Department of Spine Surgery, Dongguan Tungwah Hospital, Dongguan, China

**Keywords:** Dietary inflammatory index, depression, suicide ideation, older adults, NHANES

## Abstract

**Background:**

The relationship between diet and psychological disorders in older adults has attracted considerable attention as the global trend of aging. This study examines the relationship between Dietary inflammatory index (DII) and the risk of depression and suicide in older adults using the National Health and Nutrition Examination Survey (NHANES) as a large cross-sectional study.

**Methods:**

The data were extracted from NHANES from 2005 to 2018, and cross-sectional studies were conducted on older adults (age ≥ 60 years). According to their median DII, participants were classified into High-DII (DII ≥ 1.23) and Low-DII (DII < 1.23) groups. Depression was the primary outcome, and suicidal ideation was a secondary outcome. Utilizing multi-factor logistic regression to correlate DII with outcomes.

**Results:**

There were 10,956 elderly participants included in the analysis. In comparison to Low-DII group, High-DII group exhibited a higher rate of depression (8.9% vs. 6.7%; *P* < 0.001) and higher ideation to commit suicide (3.7% vs. 3.0%; *P* = 0.039). Moreover, in terms of gender ratio, men accounted for 44% of the High-DII group, which was significantly lower than 56.2% of the Low-DII group (*P* < 0.001). Furthermore, logistic regression revealed that High-DII group had a higher risk of depression in the previous 2 weeks (OR = 1.358, 95% CI: 1.180–1.564; *P* < 0.001) and a higher risk of suicidal ideation (OR = 1.244, 95% CI: 1.010–1.532; *P* = 0.040). Additionally, after adjusting for demographic covariates such as age, gender and race, High-DII group still had a higher risk of depression (OR = 1.293, 95% CI: 1.121–1.493; *P* < 0.001) and suicidal ideation (OR = 1.261, 95% CI: 1.021–1.55; *P* = 0.031). Furthermore, after adjusting for various covariates like demographic, social factors, and comorbidities, the High-DII group remained at higher risk for depression (OR = 1.178, 95% CI: 1.019–1.363; *P* = 0.027), and the risk of comorbid suicidal ideation remained high (OR = 1.136, 95% CI: 0.917–1.408), but the difference was not significant (*P* = 0.243).

**Conclusion:**

In older adults, high levels of DII are associated with depression and suicidal ideation. Multiple factors affect the mental health of older adults, and it is unknown to what extent a pro-inflammatory diet contributes to depression and suicidal thoughts in older adults. Nonetheless, daily dietary management in older adults should be emphasized.

## Introduction

The mental health of the elderly is gradually becoming a critical global public health issue as the global aging trend develops. As of 2013, nearly 10% of the world’s population (approximately 615 million) suffers from a psychiatric disorder, with more than 20% of the older population over the age of 60 suffering from a psychiatric disorder, of which depression is one of the most common (1). Many factors, including widowhood, living alone, reduced intergenerational communication, declining physical function, and various chronic diseases, are increasing the elderly’s mental health risks, seriously impacting their daily lives, and lowering their quality of life in their later years (2, 3). The most frequent mental illness in the elderly is depression (4). Research conducted in Germany (5). According to a population-based survey, it is reported that 28.7% of older adults had depressive symptoms, among them, 6.6% were severe depression, and the older the person, the higher the prevalence of depressive symptoms. Another study (6) conducted in Greece found that 34.4% of older adults had impaired cognitive functions, and 32.3% experienced depressive symptoms. In addition, a large cross-sectional study (7) from China indicated that the prevalence of somatic symptom disorders in older adults was 63.2%, significantly higher than in the non-elderly population (45.3%); similarly, the risk of depression or anxiety was 3.7 times that of the general population. Furthermore, patients with chronic depressive symptoms are prone to suicidal thoughts, with up to 15% of depressed patients reportedly preferring suicide ideation (8).

There is research evidence that depression is significantly associated with chronic systemic inflammation (9). Chronic inflammation can mediate a permanent reorganization of inflammatory neurotransmitter pathways, resulting in the transition from acute to chronic pain and promoting depression, anxiety, and sleep disturbances (10–12). The association between diet and chronic systemic inflammation is quite close, and an inappropriate dietary structure or pattern is a significant source of chronic systemic inflammation in the organism (13). Dietary inflammatory index (DII) is a novel tool for assessing the subversive potential of the diet and the amount of pro-inflammatory food components in an individual’s dietary composition (14). DII is strongly associated with mental illness, compared to controls, patients with schizophrenia appeared to have higher DII scores (1.99 ± 1.39 vs. 1.60 ± 1.38; *P* = 0.009), and each unit increase in DII score was associated with a 62% increase in the odds of developing schizophrenia (OR = 1.62; 95% CI 1.17–2.26) (14). Chronic systemic inflammation can significantly increase with high levels of DII (15). Several studies (16, 17) have found that pro-inflammatory dietary patterns are significantly associated with an elevated risk of depression in adults, as high levels of DII increase the risk of depressive symptoms. Nonetheless, these studies have been limited to specialized populations, like medical personnel. However, these were only single-center studies on specific people, such as medical personnel (18). Furthermore, the relationship between suicide and DII has not been clarified.

There is a scarcity of direct and robust evidence between DII to an increased risk of depression and suicide in the elderly population. Data from large-scale demographic surveys are still required, given the potential economic burden and adverse effects of psychological disorders in older adults on an individual and societal level and the guiding significance of a rational daily dietary pattern. It is essential to investigate the relationship between psychological disorders and nutritional habits in the elderly, emphasizing daily dietary interventions.

## Materials and Methods

### Included Population

The National Health and Nutrition Examination Survey (NHANES) is a cross-sectional survey conducted by the National Center for Health Statistics and the Centers for Disease Control and Prevention, utilizing data from a nationally representative sample of the U.S. civilian population. This study’s dataset was constructed using NHANES public data files from 2005 to 2018, and the study population comprised all NHANES respondents.

### Dietary Inflammatory Index Evaluation and Grouping

The key exposure variable in this study was DII, and the types and amounts of food and beverages consumed by the participants in 24 h were extracted. As documented in the literature (19), DII was calculated and briefly explained as follows: DII for each nutrient or dietary ingredient = [(daily intake of that nutrient or dietary ingredient - global per capita daily intake of that nutrient or dietary ingredient)/that nutrient or dietary ingredient Standard deviation of global per capita daily intake] × inflammatory effect index of that nutrient or dietary ingredient, and the sum of DII of each nutrient or dietary ingredient was the total DII score of individual study subjects (see Supplementary Table 1 for specific nutrients and their inflammation indexes). According to the median DII of all included subjects, the participating population was classified into a High-DII group (DII ≥ median) and a Low-DII group (DII < median).

### Outcomes

This study’s primary outcome was depression. NHANES questionnaire yielded Patient Health Questionnaire-9 (PHQ-9) data. They consisted of nine clinical depression symptom items for the past 2 weeks, with scores ranging from zero to three for each item, with zero indicating no symptoms during the period, one suggesting a few days of symptoms, two showing more than half of the days with symptoms, and three describing symptoms almost every day. The cumulative PHQ-9 score was used to determine the presence and severity of clinical depressive symptoms in the previous 2 weeks. According to prior studies (20), when the total PHQ-9 score = 10, there was good sensitivity and specificity for major depression. Therefore, the study participant population with PHQ-9 < 10 was considered non-depressed patients and PHQ-9 ≥ 10 as depressed patients.

Even though the proportion of elderly suicides is relatively low compared to depressive symptoms, it is important to focus on the study of suicidal ideation in the elderly, considering that suicide is mainly secondary to severe depressive symptoms and the heavy burden on society. Consequently, suicidal ideations were chosen as the secondary outcome in this study, and item nine of the PHQ-9 (Scoring rules are in Supplementary Table 2) explicitly asked respondents if they had had suicidal thoughts in the past 2 weeks, which was demonstrated to be a valid predictor of future attempted or completed suicide (21).

### Covariates

Covariates were chosen to influence depression-related factors that have previously been reported. Age, gender, and race, for example, were included as demographic characteristics. Social factors include education level, marital status, household income up to the poverty level, health insurance coverage, and data on everyday health-related behaviors such as smoking and alcohol consumption. In addition, medical comorbidity variables such as Body mass index (BMI), diabetes, and cancer were collected.

### Data Analysis

Quantitative data were examined using the *t*-test or Analysis of Variance, and data for categorical variables were analyzed using the χ2 test for differences in cohort characteristics between exposure groups. The preliminary analysis employed multi-factor logistic regression to determine the relationship between the exposure group and the outcome variable. All descriptive studies were assessed for significance using two-sided tests at the *P* < 0.05 level of energy. Eventually, a Generalized additive model was employed to test for non-linear relationships between the outcome variables and exposure factors. All data analyses were performed utilizing Empower Stats software^1^ (X&Y solutions, Inc., Boston MA, United States) and R.3.5.2.^2^

## Results

**FIGURE 1:**
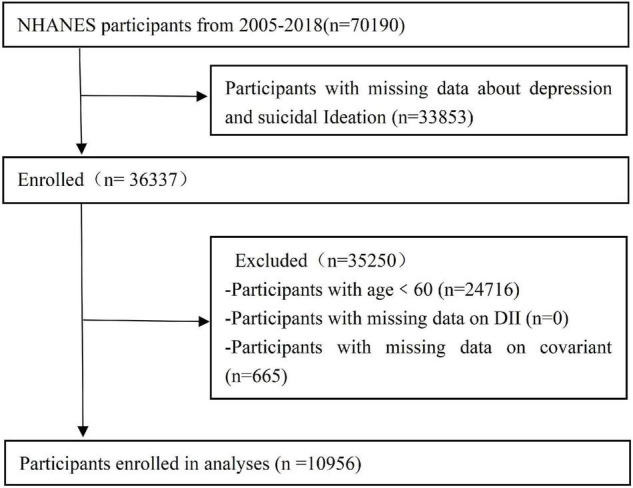
Flow diagram of the screening and selection process.

First, initial survey data were obtained from 70,190 participants. After eliminating individuals who lacked outcome, exposure, or covariate data (with the exceptions noted above), the analysis included 10,956 older participants (Figure 1).

### Essential Characteristics of Participants in High-Dietary Inflammatory Index and Low-Dietary Inflammatory Index Groups

All elderly subjects’ median DII was calculated to be 1.23. As a result, the subjects were classified into the High-DII group (DII ≥ 1.23) and Low-DII group (DII < 1.23), with 5,478 participants in each group. Table 1 depicts the distribution of cohort characteristics stratified by DII level. In the preliminary analysis, the average age of participants in the High DII group was (70.0 ± 7.0) years, whereas the average age of participants in the Low-DII group was (69.9 ± 7.0) years. There was no statistically significant difference between the groups (*P* > 0.05). Furthermore, regarding gender ratio, males accounted for 44% of the High-DII group, significantly lower than 56.2% of the Low-DII group (*P* < 0.001), indicating that men tend to have a lower DII. Similarly, in terms of social factors, the High-DII group had a higher proportion ratio of family income to poverty ≤ 2.9,” “Married or living with a partner,” and “Never married” compared to the Low-DII group (*P* < 0.05). Furthermore, the differences in BMI, hypertension, diabetes and tumor history were statistically significant (*P* < 0.05).

**TABLE 1:** Characteristics of participants enrolled in the study from the NHANES (2005 to 2018).

Characteristic	Low-DII (DII < 1.23, *N* = 5,478)	High-DII (DII ≥ 1.23, *N* = 5,478)	*P*-value
Age (y)	69.9 ± 7.0	70.0 ± 7.0	0.587
Male sex	3080 (56.2)	2413 (44.0)	<0.001
Race			<0.001
Hispanic	3353 (61.2)	3183 (58.1)	
Non-Hispanic white	707 (12.9)	682 (12.4)	
Non-Hispanic black	928 (16.9)	1288 (23.5)	
Other	490 (8.9)	325 (5.9)	
Education beyond high school	4100 (74.8)	3603 (65.8)	<0.001
Marital status			<0.001
Never married	253 (4.6)	282 (5.1)	
Married or living with partner	3405 (62.2)	3006 (54.9)	
Divorced, separated, or widowed	1820 (33.2)	2190 (40.0)	
Ratio of family income to poverty level			<0.001
<1.0	1229 (22.4)	1490 (27.2)	
1.0∼2.9	2200 (40.2)	2523 (46.1)	
≥3	2049 (37.4)	1465 (26.7)	
Health insurance coverage	5099 (93.1)	4987 (91.0)	<0.001
BMI (kg/m2)			<0.001
<25	1517 (27.7)	1322 (24.1)	
25∼29	2004 (36.6)	1938 (35.4)	
≥30	1957 (35.7)	2218 (40.5)	
eGFR, ml/min per 1.73 m^2^	75.0 ± 18.4	74.5 ± 19.0	0.120
ALT (U/L)	23.0 ± 16.1	21.6 ± 13.7	<0.001
AST (U/L)	25.4 ± 12.0	24.9 ± 12.0	0.038
HbA1c	6.0 ± 1.1	6.1 ± 1.2	<0.001
Alcohol user	3250 (59.3)	2643 (48.2)	<0.001
Smoker	2811 (51.3)	2830 (51.7)	0.716
Hypertension	3769 (68.8)	4061 (74.1)	<0.001
Cancer	1150 (21.0)	1049 (19.1)	0.016
Diabetes	1756 (32.1)	1987 (36.3)	<0.001
Suicidal ideation	166 (3.0)	205 (3.7)	0.039
Depressed	367 (6.7)	487 (8.9)	<0.001

*All values are displayed as n (%). χ^2^ analysis is used to test significance between groups for categorical variables. BMI, body mass index; eGFR, estimated glomerular filtration rate; HbA1c, glycosylated Hemoglobin, type A1C; ALT, alanine aminotransferase; AST, aspartate aminotransferase.*

### The Relationship Between Dietary Inflammatory Index and Depression and Suicidal Ideation

#### Both Depression and Suicidal Ideation Were Higher in the High-Dietary Inflammatory Index Group Than in the Low-Dietary Inflammatory Index Group Participants

The High-DII group had 487 (8.9%) participants with comorbid depression and 205 (3.7%) with comorbid suicidal ideation, while the Low-DII group had 367 (6.7%) participants with comorbid depression and 166 (3.0%) with comorbid suicidal ideation. Therefore, depression and suicidal ideation were higher in the High-DII group than in the Low-DII group, and the differences were statistically significant (*P* < 0.05).

#### Dietary Inflammatory Index Is a Sole Risk Factor for Depression and Suicidal Ideation

As indicated in Table 2, in an unadjusted multivariate logistic regression analysis, high DII was an independent risk factor for depression (OR = 1.358, 95% CI: 1.180–1.564; *P* < 0.001) and suicidal ideation (OR = 1.244, 95% CI: 1.010–1.532; *P* = 0.040) in the previous 2 weeks. After adjusting for demographic covariates like age, sex, and race, high DII was still associated with a higher risk of depression (OR = 1.293, 95% CI: 1.121–1.493; *P* < 0.001) and suicidal ideation (OR = 1.261, 95% CI: 1.021–1.55; *P* = 0.031). In addition, after adjusting for various covariates like demographic, social factors, and comorbidities, high DII remained an independent risk factor for depression (OR = 1.178, 95% CI: 1.019–1.363; *P* = 0.027). However, it was not a risk factor for suicidal ideation (OR = 1.136, 95% CI: 0.917–1.408; *P* = 0.243).

**TABLE 2:** Multi-factor logistic regression analysis for associations DII high-level and outcomes.

	Depressed		Suicidal ideation	
DII high-level	OR (95% CI)	*P*-value	OR (95% CI)	*P*-value
Un- adjusted	1.358 (1.180–1.564)	<0.001	1.244 (1.010–1.532)	0.040
Model 1	1.293 (1.121–1.493)	<0.001	1.261 (1.021–1.557)	0.031
Model 2	1.191 (1.030–1.377)	0.018	1.182 (0.955–1.462)	0.124
Model 3	1.178 (1.019–1.363)	0.027	1.136 (0.917–1.408)	0.243

*Model 1 was adjusted for age, sex, and race.*

*Model 2 was adjusted for age, sex, race, education level, marital status, ratio of family income to poverty level, and health insurance coverage.*

*Model 3 was adjusted for age, sex, race, education level, marital status, family income to poverty level, health insurance coverage, BMI, smoker, alcohol user, hypertension, cancer, and diabetes.*

Moreover, subgroup logistic regression analyses by gender revealed that women with high DII were more likely to have comorbid suicidal ideation (OR = 1.355, 95% CI: 1.004–1.829; *P* = 0.047), while men were not (Table 3).

**TABLE 3:** Subgroup multi-factor logistic regression analysis for the association between DII high-level and outcomes.

	Depressed			Suicidal ideation		
	OR (95%CI)	*P*-value	Interaction *P*-value	OR (95%CI)	*P*-value	Interaction *P*-value
Sex			0.374			0.409
Female	1.219 (1.013–1.467)	0.036		1.355 (1.004–1.829)	0.047	
Male	1.399 (1.123–1.744)	0.003		1.135 (0.843–1.527)	0.403	
BMI			0.306			0.237
<25	1.372 (1.022–1.842)	0.035		1.316 (0.872–1.987)	0.192	
25∼29	1.549 (1.189–2.020)	0.001		1.520 (1.058–2.184)	0.023	
≥30	1.196 (0.976–1.466)	0.084		1.007 (0.728–1.393)	0.967	
Alcohol user			0.277			0.558
Yes	1.145 (1.147–1.747)	0.001		1.283 (0.945–1.743)	0.110	
No	1.028 (0.998–1.463)	0.053		1.132 (0.851–1.507)	0.394	
Smoker			0.606			0.410
Yes	1.315 (1.092–1.585)	0.004		1.160 (0.889–1.512)	0.274	
No	1.418 (1.142–1.760)	0.002		1.389 (0.991–1.946)	0.057	
Hypertension			0.833			0.217
Yes	1.328 (1.130–1.562)	<0.001		1.143 (0.898–1.455)	0.278	
No	1.376 (1.031–1.838)	0.030		1.542 (1.022–2.327)	0.039	
Cancer			0.421			0.786
Yes	1.530 (1.105–2.118)	0.010		1.184 (0.767–1.828)	0.445	
No	1.319 (1.128–1.543)	<0.001		1.268 (1.000–1.608)	0.050	
Diabetes			0.878			0.599
Yes	1.343 (1.088–1.657)	0.006		1.306 (0.946–1.803)	0.104	
No	1.313 (1.084–1.590)	0.005		1.166 (0.887–1.534)	0.271	

#### Dose-Response Relationship Between Dietary Inflammatory Index and the Risk of Depression and Suicide in the Elderly

**FIGURE 2:**
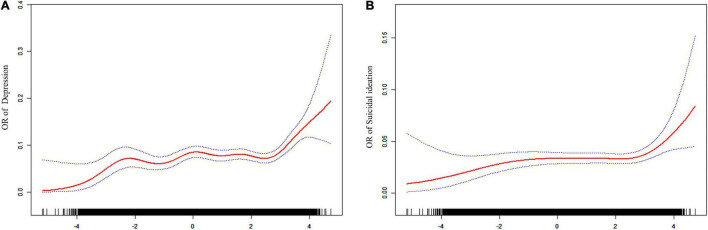
Dose-response association between **(A,B)** OR of depression and suicidal ideation and DII (adjusted for age, sex, race, smoker, alcohol user, glucocorticoid user, hypertension, cancer, and diabetes).

Figure 2 depicts the dose-response relationship between DII and outcome indicators. DII was positively associated with the risk of depressive symptoms, and the overall risk of depression in older adults tended to increase progressively with increasing DII (Figure 2A). Similarly, the relationship between DII and the risk of comorbid suicidal ideation also increased progressively with DII levels (Figure 2B).

## Discussion

Using a nationally representative cross-sectional study of older adults, this study demonstrated a significant relationship between high levels of DII and depression in older adults and a relationship with suicidal ideation in the past 2 weeks.

These outcomes are consistent with earlier studies (16) on the relationship between diet and depression; a high intake of pro-inflammatory foods significantly increases the risk of depressive symptoms. Those at the highest risk for depression had significantly lower consumption of meat, fish, and eggs, higher consumption of added sugars, and relatively lower consumption of fruits, vegetables, and fiber (16, 18). Furthermore, a study of medical personnel (18) found a significant positive correlation between high PHQ-9 scores and high DII levels when gender, psychiatric diagnosis, physical activity, and mental exercise were all taken into account (*P* < 0.01). Another study (22) of residential female undergraduates discovered that higher DII was significantly associated with an increased likelihood of stress symptoms (OR = 1.41, 95% CI: 1.12–1.77; *P* = 0.003) and anxiety symptoms (OR = 1.35, 95% CI: 1.07–1.69; *P* = 0.01). The relationship between dietary patterns and depression remains significant, and strong epidemiological evidence proposes that poor diet may hurt mental health disorders (23). However, after adjusting for marriage, education, and family poverty to income ratios, the significance of DII concerning the risk of suicide in older patients decreased significantly. Diet may have a lesser impact on suicidal ideation than social factors. Indeed, according to earlier surveys, unhappy marriages, inadequate education, and economic poverty are significant social factors contributing to older adults’ suicide (24, 25).

Furthermore, our gender stratification results indicate that both men and women in the High-DII group are at higher risk of developing depression than those in the Low-DII group. Women with high DII are more likely to experience suicidal ideation. The differential results for suicidal ideation may be driven primarily by the female population. Although women generally have lower rates of committing suicide than men, they have higher suicidal ideation and attempt rates, especially in women over the age of 75 (26). This could be related to the unstable nature of the female elderly, who are emotional in situations and have weak resistance to stress and a relative lack of social support. When confronted with unexpected situations, they are more prone to depression and anxiety symptoms (27, 28).

In short, the relationship between diet and mental health is complicated. Even though our observational studies reveal that high levels of DII may be related to depressive mood and suicidal ideation, there are many biological, psychological and social factors involved. As the global trend of population aging develops, numerous aspects of demographic changes increase socioeconomic pressures, and declining somatic functionality further complicates the discussion linked to mental health in the elderly population. The previous study has focused on the association between social factors and mental health in older adults.

However, there are some limitations of the present work. First, it is difficult to elaborate on the causal relationship between an inflammatory diet and depression/suicide in the present cross-sectional study. Because the NHANES study collected data at a single time point, nutritional data were recorded only once for all participants, and PHQ-9 scores, as well as suicidal ideation questionnaires, were measured only once, which resulted in some possible bias in DII scores and PHQ-9 scores. In addition, this study may also have overlooked some relevant factors that influence depression/suicidal ideation in older adults, such as the presence of major life changes, chronic pain, family atmosphere, etc. Therefore, in the future, we need to conduct a multicenter longitudinal clinical trial to confirm our findings, dynamically assess changes in each of the factors that may influence depression/suicidal ideation in older adults, and conduct a long follow-up to investigate how inflammatory diet specifically affects the onset and progression of depression in older adults.

Nonetheless, studies of dietary patterns and cognitive problems in older adults are scarce and primarily single-centered with small sample sizes. As our understanding of the factors influencing mental health in the elderly population improves, we should carefully evaluate dietary patterns’ long-term benefits and harms to mental disease in future studies and develop appropriate nutritional patterns for the elderly.

## Conclusion

By analyzing a nationally representative sample, we discovered that high levels of DII were related to moderate to severe clinical depression and suicidal ideation in older adults. This relationship is complicated, and further studies are required to understand it fully. However, the extent to which a pro-inflammatory diet leads to significant depressive symptoms and suicidal ideation is complicated and requires further research to demonstrate this relationship.

## Data Availability Statement

Publicly available datasets were analyzed in this study. This data can be found here: https://www.cdc.gov/nchs/nhanes/index.htm.

## Ethics Statement

Before being interviewed or examined, all survey participants provided informed consent, and the NCHS Ethics Review Board approved the data collection protocol.

## Author Contributions

YX and WH carried out the acquisition and interpretation of data and was the major contributor to drafting the manuscript and participated in drawing tables and diagrams. YX carried out the clinical data collection and analysis. WH contributed to the ideas of the article and reviewed the manuscript. Both authors provided final approval for publishing the manuscript.

## Conflict of Interest

The authors declare that the research was conducted in the absence of any commercial or financial relationships that could be construed as a potential conflict of interest.

## Publisher’s Note

All claims expressed in this article are solely those of the authors and do not necessarily represent those of their affiliated organizations, or those of the publisher, the editors and the reviewers. Any product that may be evaluated in this article, or claim that may be made by its manufacturer, is not guaranteed or endorsed by the publisher.

## Footnotes

1
www.empowerstats.com

2
http://www.R-project.org

## Supplementary Material

Supplementary Table 1 Forty five nutrients included in DII calculation.

Supplementary Table 2 PHQ-9 score.

## Abbreviations

DII: dietary inflammatory index
NHANES: National Health and Nutrition Examination Survey
PHQ-9: patient health questionnaire-9
BMI: body mass index.
