# Supplementary material for: Association of Dietary Inflammatory Index With Depression and Suicidal Ideation in Older Adult: Results From the National Health and Nutrition Examination Surveys 2005–2018
Source: Front Psychiatry. 2022 Jul 5;13:944154. doi: 10.3389/fpsyt.2022.944154 (PMC9294216; doi:10.3389/fpsyt.2022.944154)
Supplement: Supplementary Table 1 — Forty five nutrients included in DII calculation. [file Table_1.DOCX]

| **Items** | **Nutrients (inflammation index)** |
| --- | --- |
| **Proinflammatory nutrient (9)** | Saturated grease (0.373), Total fat (0.298), Trans fat (0.229), Energy (0.180), Cholesterol (0.110), Vitamin (B12 0.106), Carbohydrate (0.097), Iron (0.032), and Protein (0.021) |
| **Anti inflammatory nutrient/food (36)** | Alcohol (-0.278), Onion (-0.301), Zinc (-0.313), Vitamin A (-0.401), Polyunsaturated fatty acids (-0.337), Garlic (-0.412), Vitamin B6 (-0.365), Flavanones (-0.250), Catechin (-0.415), Monounsaturated fatty acids (-0.009), Vitamin E (-0.419), Rosemary (-0.013), Vitamin C (-0.424), Vitamin B2 (-0.068), Omega-3 fatty acids (-0.436), Vitamin B1 (-0.098), Vitamin D (-0.446), Thyme / oregano (-0.102), Ginger (-0.453), Caffeine (-0.110), Flavonoids (-0.467), Pepper (-0.131), Magnesium (-0.484), Anthocyanin (-0.131), Green / black tea (-0.536), Saffron (-0.140), β-Carotene (-0.584), Eugenol (-0.140), Isoflavone (-0.593), Omega-6 fatty acids (-0.159), Flavonoids (-0.616), Folic acid (-0.190), Fiber (-0.663), Selenium (-0.191), Turmeric (-0.785), Vitamin B3 (-0.246) |

**45 nutrients included in DII calculation**

**Note:** the higher the inflammation index is, the higher the pro-inflammatory effect of the nutrients taken is. The lower the inflammation index (the negative value), the better the anti-inflammatory effect of the nutrients taken is.
