# Supplementary material for: Association of Dietary Inflammatory Index With Depression and Suicidal Ideation in Older Adult: Results From the National Health and Nutrition Examination Surveys 2005–2018
Source: Front Psychiatry. 2022 Jul 5;13:944154. doi: 10.3389/fpsyt.2022.944154 (PMC9294216; doi:10.3389/fpsyt.2022.944154)
Supplement: Supplementary Table 2 — PHQ-9 score. [file Table_2.DOCX]

| **PHQ-9 items** | **Scores** | | | |
| --- | --- | --- | --- | --- |
|  | None | A few days | More than half the time | Almost every day |
| 1. Little interest or pleasure in doing things | 0 | 1 | 2 | 3 |
| 2. Feeling down, depressed, or hopeless | 0 | 1 | 2 | 3 |
| 3. Trouble falling or staying asleep, or sleeping too much | 0 | 1 | 2 | 3 |
| 4. Feeling tired or having little energy | 0 | 1 | 2 | 3 |
| 5. Poor appetite or overeating | 0 | 1 | 2 | 3 |
| 6. Feeling bad about yourself or that you are a failure | 0 | 1 | 2 | 3 |
| 7. Trouble concentrating on things | 0 | 1 | 2 | 3 |
| 8. Moving or speaking so slowly that other people have noticed | 0 | 1 | 2 | 3 |
| 9. Thoughts that you would be better off dead or of hurting yourself | 0 | 1 | 2 | 3 |
